# Comparative Activities of Novel Therapeutic Agents against Molecularly Characterized Clinical Carbapenem-Resistant *Enterobacterales* Isolates

**DOI:** 10.1128/spectrum.01002-23

**Published:** 2023-05-15

**Authors:** Jocelyn Qi-Min Teo, Hong Yi Chang, Si Hui Tan, Cheng Yee Tang, Rick Twee-Hee Ong, Karrie Kwan Ki Ko, Shimin Jasmine Chung, Thuan Tong Tan, Andrea Lay-Hoon Kwa

**Affiliations:** a Department of Pharmacy, Singapore General Hospital, Singapore, Singapore; b Department of Clinical Translational Research, Singapore General Hospital, Singapore, Singapore; c Saw Swee Hock School of Public Health, National University of Singapore and National University Health System, Singapore, Singapore; d Department of Microbiology, Singapore General Hospital, Singapore, Singapore; e Department of Infectious Diseases, Singapore General Hospital, Singapore, Singapore; f Singhealth Duke-NUS Medicine Academic Clinical Programme, Singapore, Singapore; g Emerging Infectious Diseases, Duke-National University of Singapore Medical School, Singapore, Singapore; University of Arizona/Banner Health

**Keywords:** carbapenem resistant, *Enterobacterales*, antibiotic resistance, susceptibility testing, ceftazidime-avibactam, imipenem-relebactam, meropenem-vaborbactam, eravacycline, plazomicin

## Abstract

Limited treatment options exist for the treatment of carbapenem-resistant *Enterobacterales* (CRE) bacteria. Fortunately, there are several recently approved antibiotics indicated for CRE infections. Here, we examine the *in vitro* activity of various novel agents (eravacycline, plazomicin, ceftazidime-avibactam, imipenem-relebactam, and meropenem-vaborbactam) and comparators (tigecycline, amikacin, levofloxacin, fosfomycin, polymyxin B) against 365 well-characterized CRE clinical isolates with various genotypes. Nonduplicate isolates collected from the largest public health hospital in Singapore between 2007 and 2020 were subjected to antimicrobial susceptibility testing (broth microdilution or antibiotic gradient test strips). Susceptibilities were defined using Clinical and Laboratory Standards Institute (CLSI) or Food and Drug Administration (FDA) interpretative criteria. Sequence types and resistance mechanisms were characterized using short-read whole-genome sequencing. Overall, tigecycline and plazomicin exhibited the highest susceptibility rates (89.6% and 80.8%, respectively). However, the tigecycline susceptibility breakpoint utilized here may be outdated in view of prevailing pharmacokinetic-pharmacodynamic (PK/PD) data. Susceptibility varied by carbapenemase genotype; the β-lactam/β-lactamase inhibitor combinations were equally active (92.3 to 99.2% susceptible) against KPC producers, but only ceftazidime-avibactam retained high susceptibility (98.7%) against OXA-48-like producers. Against metallo-β-lactamase producers, only plazomicin exhibited moderate activity (77.0% susceptible). Aminoglycoside activity was also influenced by carbapenemase genotypes. This work provides an insight into the comparative activity and presumptive utility of novel agents in this geographic region.

**IMPORTANCE** This study determined the susceptibilities of carbapenem-resistant *Enterobacterales* isolates to various novel antimicrobial agents (ceftazidime-avibactam, imipenem-relebactam, meropenem-vaborbactam, eravacycline, and plazomicin). Whole-genome sequencing was performed for all strains. Our study findings provide insights into the comparative activities of novel agents in this geographic region. Plazomicin and ceftazidime-avibactam exhibited the lowest nonsusceptibility rates and may be considered promising agents in the management of carbapenem-resistant *Enterobacterales* infections. We note also that antibiotic activity is influenced by genotypes and that understanding the geographic region’s molecular epidemiology could aid in the definition of the presumptive utility of novel agents and contribute to antibiotic decision-making.

## INTRODUCTION

Resistance to carbapenems, a class of broad-spectrum antibiotics, has emerged in *Enterobacterales*, leaving very limited treatment options available for infections caused by these bacteria (1). Fortunately, several new agents have been introduced in the recent years to the ailing antibiotic pipeline to combat carbapenem-resistant *Enterobacterales* (CRE); these agents include the β-lactam/β-lactamase inhibitor (BLBLI) combinations (ceftazidime-avibactam, imipenem-relebactam, and meropenem-vaborbactam), eravacycline (glycylcycline class), and plazomicin (aminoglycoside) (2–6).

Many large-scale studies have established the broad activity of these new antibiotics against CRE; however, geographical variation in susceptibilities could exist (7). It is important to establish regional epidemiological patterns to determine the agents’ utility and place of therapy. Furthermore, emerging resistance to new agents has been reported (8). At times, resistance has been detected even prior to the introduction of the antibiotic into clinical practice (9).

Singapore’s strategic location has established the country as an international trade, travel, and medical tourism hub. The country’s strong health care infrastructure makes it a suitable antimicrobial surveillance sentinel site for the region. In view of the scarcity of susceptibility data regarding these novel agents in Southeast Asia, we sought to determine the comparative *in vitro* activities of these novel agents against a genetically diverse collection of CRE clinical strains from this geographic region.

## RESULTS

### Isolate characteristics.

The 365 CRE isolates were represented by Klebsiella pneumoniae (*n* = 153), Escherichia coli (*n* = 93), Enterobacter cloacae (*n* = 88), Klebsiella aerogenes (*n* = 23), and *Citrobacter* species (*n* = 8); they were derived from various culture sources (blood [*n* = 92], urinary [*n* = 81], gastrointestinal/abdominal [*n* = 54], wound [*n* = 53], respiratory [*n* = 37], rectal [*n* = 33], and others [*n* = 15]). The isolates were highly diverse with 59, 47, and 48 sequence types (STs) identified among K. pneumoniae, E. coli, and E. cloacae, respectively. The collection included global high-risk clones, such as K. pneumoniae belonging to clonal group (CG) 258, CG14, CG147, and CG231; E. coli belonging to ST38, ST131, ST405, and ST410; and the E. cloacae complex belonging to ST78, ST90, ST93, and ST114.

The isolates possessed a median of 12 (range, 0 to 25) antibiotic resistance genes as characterized using the NCBI-AMRFinderPlus database and the Kleborate tool (10). In brief, genomes were screened against curated databases of acquired resistance gene alleles from major relevant antibiotic drug classes. They included aminoglycosides (e.g., aminoglycoside modifying enzymes [AMEs]), β-lactams (e.g., SHV, TEM, and CTX-M β-lactamases), carbapenems (e.g., KPC and OXA-48), fluroquinolones (e.g., *qnr*), and tetracyclines (e.g., *tet*) (https://github.com/klebgenomics/Kleborate/wiki/Antimicrobial-resistance).

Carbapenemase production was identified in 301 (82.5%) isolates (KPC, 129 [35.3%]; metallo-β-lactamases [MBLs], 74 [20.3%]; OXA-48-like, 75 [20.5%]; dual carbapenemase, 17 [4.7%]; others, 6 [1.6%]). Isolates harboring two carbapenemases involved NDM + OXA-48-like enzymes except for one where IMP + KPC cocarriage was observed. Extended-spectrum and/or Amp C β-lactamases were often encountered (82.5%), both in carbapenemase producers (*n* = 246/301, 81.7%) and noncarbapenemase producers (*n* = 55/64, 85.9%). Bioinformatics analyses suggest that resistance genes were located primarily on various plasmids, where 87 different plasmid replicons were identified in the 365 CRE isolates.

### Overall antibiotic susceptibilities for all isolates.

**FIG 1:**
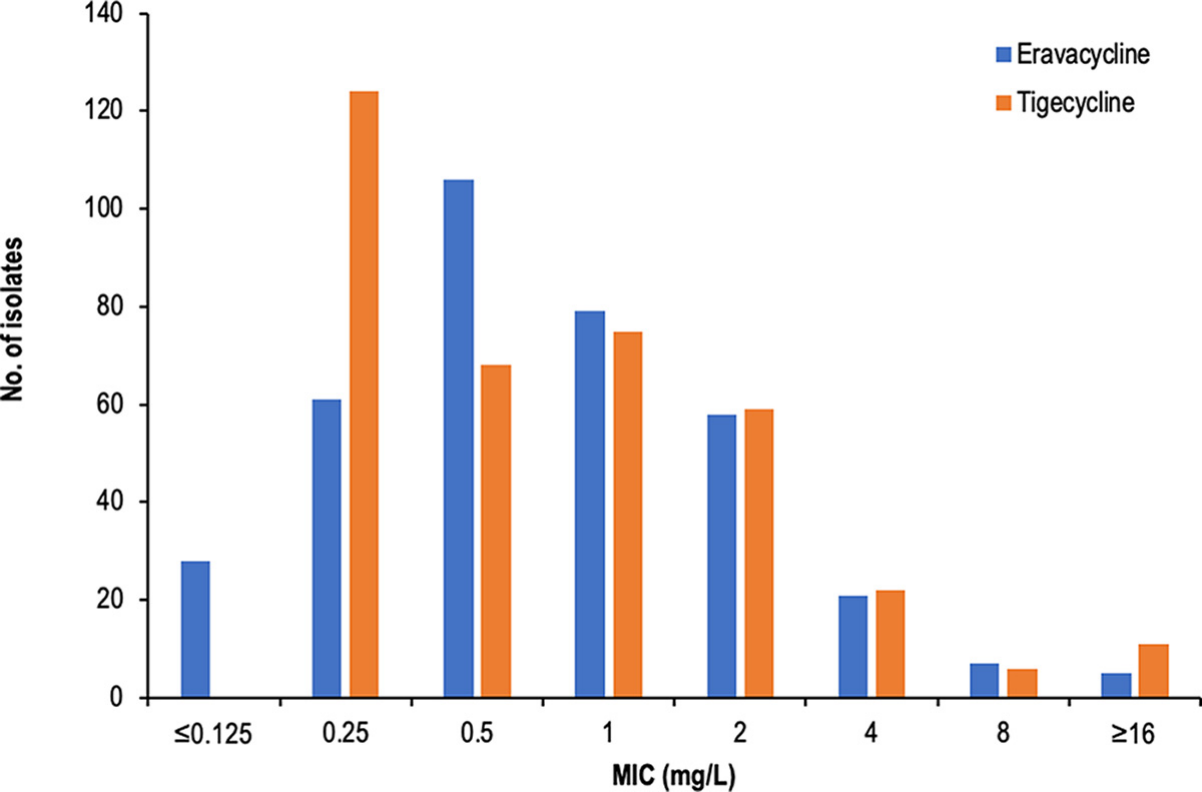
MIC distributions for eravacycline and tigecycline.

The overall antibiotic susceptibilities of the various agents are shown in Table 1. Expectedly, susceptibilities to traditional β-lactams were poor. Among all the agents, tigecycline (89.6%) and plazomicin (80.8%) exhibited the highest susceptibilities against CRE overall. A comparison of the activities of agents within the same class showed that tigecycline inhibited a larger proportion of CRE than eravacycline (89.6% versus 53.4%). However, we observed that the two agents have similar MIC distributions; MICs of the two agents were typically within one to two 2-fold dilutions of each other (Fig. 1). The MIC_50/90_ values of the two agents were also similar (tigecycline, 0.5/4 mg/L; eravacycline, 0.5/2 mg/L).

**TABLE 1:** Antibiotic susceptibilities of 365 carbapenem-resistant *Enterobacterales* isolates

Antibiotic	MIC (mg/L)			CLSI/FDA^*a*^		
	50%	90%	Range	%S	%I/SDD	%R
Ertapenem	≥32	≥32	≤1 to ≥32	0	2.2	97.8
Imipenem	16	≥32	≤0.5 to ≥32	2.7	4.4	92.9
Meropenem	≥32	≥32	≤0.5 to ≥32	6.3	5.2	88.5
Doripenem	16	≥32	≤0.5 to ≥32	7.7	8.2	84.1
Aztreonam	≥64	≥64	≤2 to ≥64	5.2	0.6	94.2
Cefepime	≥64	≥64	≤1 to ≥64	4.9	5.5	89.6
Piperacillin-tazobactam	≥128/4	≥128/4	≤4/4 to ≥128/4	1.6	0.3	98.1
Levofloxacin	16	≥64	≤0.25 to ≥64	17.5	11.5	71.0
Amikacin	≤4	≥128	≤4 to ≥128	53.7	12.9	33.4
Plazomicin	0.5	≥512	≤0.25 to ≥512	80.8	0.3	18.9
Tigecycline^*b*^	0.5	4	≤0.25 to ≥16	89.6	5.7	4.7
Eravacycline^*b*^	0.5	2	≤0.125 to ≥16	53.4		46.6
Fosfomycin^*c*^	32	≥2,048	≤0.25 to ≥2,048			
Polymyxin B	0.5	≥16	≤0.25 to ≥16		77.8	22.2
Ceftazidime-avibactam^*d*^	≤1/4	4/4	≤1/4 to ≥128/4	94.9		5.1
Imipenem-relebactam^*d*^	0.5/4	8/4	≤0.125/4 to ≥128/4	70.1	8.4	21.5
Meropenem-vaborbactam^*d*^	0.5/8	32/8	≤0.125/8 to ≥128/8	80.3	2.9	16.8

a S, susceptible; I, intermediate; SDD, susceptible-dose dependent; R, resistant.

b FDA breakpoints were utilized in place of CLSI breakpoints.

c Susceptibility categories not reported as breakpoints are applicable only for Escherichia coli.

d Only assessed for the 274 non-metallo-β-lactamase producers.

Within the aminoglycoside class, plazomicin exhibited a marked improvement in susceptibility over amikacin (80.8% versus 53.7%). Plazomicin restored susceptibility in 99/169 (58.6%) amikacin nonsusceptible isolates. Furthermore, we noted that *aac*(6′)-Ib-type genes encoding aminoglycoside modifying enzymes (AMEs) were observed in 214 (58.6%) isolates. Of these AMEs, plazomicin inhibited 163 of 164 isolates where 16S rRNA methyltransferases (16S RMTases) were absent. Resistance in the majority of the plazomicin nonsusceptible isolates (*n* = 68/70) can be explained by the presence of at least one 16S RMTase genes. The 16S RMTase genes detected were *armA* (*n* = 34), *rmtF* (*n* = 21), *rmtB* (*n* = 17), and *rmt*C (*n* = 6).

The BLBLIs exhibited differential activities against non-MBL-producing CRE isolates, with ceftazidime-avibactam being the most active (94.9%), followed by meropenem-vaborbactam (80.3%) and imipenem-relebactam (70.1%). Given that the prevalence of MBLs in local CRE isolates is approximately 20%, the overall susceptibilities of BLBLIs against CRE here will be at best moderate (~50 to 75%).

We also compared the antibiotic susceptibilities of the three predominant CRE species (Table 2). Among the three species, E. coli displayed the highest antibiotic susceptibilities with all of the novel agents and last-line comparators inhibiting >80% of the tested isolates. In contrast, K. pneumoniae isolates displayed substantially lower susceptibility rates for most of the antibiotics. Notably, polymyxin B was the least active against E. cloacae (only 56% exhibited MICs of ≤2mg/L); this finding is contributed partly by the higher prevalence of *mcr* in Enterobacter cloacae (19 of 26 *mcr*-positive isolates) than that in the other two species.

**TABLE 2:** Antibiotic susceptibilities of CRE isolates belonging to predominant species^*a*^

Antibiotic	Data by species					
	K. pneumoniae (*n* = 153)		E. coli (*n* = 93)		E. cloacae (*n* = 88)	
	MIC_50/90_ (mg/L)	S (%)	MIC_50/90_ (mg/L)	S (%)	MIC_50/90_ (mg/L)	S (%)
Ertapenem	≥32/≥32	0	≥32/≥32	0	≥32/≥32	0
Imipenem	16/≥32	3.3	16/≥32	1.1	16/≥32	4.5
Meropenem	≥32/≥32	0.7	16/≥32	16.1	16/≥32	6.8
Doripenem	16/≥32	2.6	8/≥32	16.1	16/≥32	7.9
Aztreonam	≥64/≥64	1.3	≥64/≥64	7.5	≥64/≥64	9.1
Cefepime	≥64/≥64	1.3	≥64/≥64	5.4	≥64/≥64	11.4
Piperacillin-tazobactam	≥128/≥128	0	≥128/≥128	0	≥128/≥128	5.7
Levofloxacin	32/≥64	11.1	32/≥64	12.9	1/32	25.0
Amikacin	16/≥128	36.0	≤4/32	64.5	≤4/32	65.9
Plazomicin	1/≥512	63.4	1/2	94.6	0.5/2	90.9
Tigecycline^*b*^	1/4	82.4	≤0.25/1	95.7	0.5/2	95.5
Eravacycline^*b*^	1/4	29.4	0.25/1	83.9	0.5/2	61.4
Fosfomycin^*c*^	64/≥2,048		2/≥2,048	84.9	32/1,024	
Polymyxin B	0.5/≥16		0.5/1		2/≥16	
Ceftazidime-avibactam^*d*^	≤1/4	95.0	≤1/8	92.9	≤1/2	98.1
Imipenem-relebactam^*d*^	1/8	51.2	0.5/4	82.9	0.5/2	84.9
Meropenem-vaborbactam^*d*^	1/64	64.5	0.5/4	91.4	≤0.125/0.5	96.2

a Less common species (Klebsiella aerogenes and *Citrobacter* spp.) were not included here. *n* = 334.

b FDA breakpoints were utilized in place of CLSI breakpoints.

c Susceptibility categories reported only for Escherichia coli.

d Only assessed for the non-metallo-β-lactamase producers.

### Effect of carbapenemase genotypes on susceptibility.

Table 3 lists the susceptibility of the various antibiotics against isolates with specific carbapenemase genotypes. Against KPC producers, tigecycline, plazomicin, and all three new BLBLIs inhibited >90% of the isolates. Against OXA-48-like-producers, tigecycline and ceftazidime-avibactam inhibited >90% of the isolates. Only tigecycline inhibited >90% of MBL-producing isolates. Cocarriage of two carbapenemases rendered all antibiotics ineffective in a large proportion of the isolates; the susceptibility of the tested agents ranged from 0 to 35.3%, except tigecycline (88.2%). Among the nonproducers, plazomicin (92.2%) and ceftazidime-avibactam (81.3%) were the most active agents.

**TABLE 3:** Antibiotic susceptibilities in relation to differing carbapenemase genotypes^*a*^

Antibiotic	% susceptibility of:				
	Non-CP (*n* = 64)	MBL (*n* = 74)	Dual^*b*^ (*n* = 17)	KPC (*n* = 129)	OXA-48-like (*n* = 75)
Ertapenem	0	0	0	0	0
Imipenem	10.9	1.4	0	0.8	1.3
Meropenem	12.5	1.4	0	0	18.7
Doripenem	17.2	1.4	0	2.3	16.0
Aztreonam	3.1	8.1	0	0	9.3
Cefepime	9.4	1.4	0	0.8	6.7
Piperacillin-tazobactam	1.6	1.4	0	0	0
Levofloxacin	21.9	9.5	0	27.1	6.7
Amikacin	57.8	47.3	11.8	67.4	38.7
Plazomicin	92.2	77	17.7	98.4	57.3
Tigecycline^*c*^	79.7	90.5	88.2	90.7	94.7
Eravacycline^*c*^	39.1	55.4	35.3	64.3	45.3
Ceftazidime-avibactam^*d*^	81.3			99.2	98.7
Imipenem-relebactam^*d*^	73.4			92.3	28.0
Meropenem-vaborbactam^*d*^	79.7			99.2	46.7

a A total of 6 isolates with uncommon carbapenemases (*bla*_IMI_ and *bla*_OXA-23_) were excluded here due to the small number. Fosfomycin susceptibility is not displayed here as it applies only to E. coli. Polymyxin susceptibility was not displayed here due to the lack of a “susceptible” CLSI breakpoint. CP, carbapenemase producing; MBL, metallo-β-lactamase producers.

b Refers to the cocarriage of carbapenemases: all isolates carried NDM+OXA-48-like enzymes except one isolate (IMP+KPC).

c FDA breakpoints were utilized in place of CLSI breakpoints.

d Only assessed for the non-metallo-b-lactamase producers.

## DISCUSSION

A robust armamentarium of new therapeutic agents for treating CRE isolates has been introduced into the market in recent years, lending some promise to the management of the infections they cause. Here, we assessed the *in vitro* activity of a broad range of available therapeutic agents against 365 diverse CRE isolates. Study findings here provide novel perspectives of new CRE agents in the Southeast Asian regional context.

First, the high proportion of resistance to newer agents observed among CRE isolates here highlights the continued challenge faced by physicians in managing such infections; none of the tested agents were able to inhibit >90% of the isolates. Nevertheless, we identified plazomicin as a promising addition to the CRE armamentarium, with 80.8% of isolates exhibiting susceptibility. Although aminoglycoside resistance was frequently related to the presence of 16S RMTases (*n* = 69/365, 18.9%), plazomicin displayed improved susceptibility over amikacin, inhibiting nearly 60% of amikacin nonsusceptible isolates. AMEs, which are often plasmid mediated, are the leading cause of aminoglycoside resistance. Plazomicin was designed to evade these clinically relevant AMEs, including the amikacin resistance conferring AAC(6′) enzymes. Here, we detected the presence of these enzymes in a large proportion of our isolates (*n* = 214/365, 58.6%), including in amikacin-susceptible isolates (*n* = 150). Plazomicin was able to inhibit all AAC(6′)-harboring isolates where 16S RMTases were absent, establishing its purported superiority over amikacin.

The assessment of comparative activities of tigecycline and eravacycline is confounded by the discrepancies in susceptibility breakpoints between new and old agents. The superior activity of tigecycline observed here is likely misleading due to the application of current FDA breakpoints. The FDA cited concerns about the nonlinear plasma protein binding of the agent as the reason for the lower susceptibility breakpoint for eravacycline (≤0.5 mg/L) than that for tigecycline (≤2 mg/L). However, this concern also exists for tigecycline, where it has been suggested that the current FDA tigecycline breakpoint may be too high (11, 12). In line with prevailing pharmacokinetic-pharmacodynamic (PK/PD) evidence, EUCAST has revised the susceptibility breakpoint of tigecycline to ≤0.5 mg/L, which is the same breakpoint indicated by both EUCAST and FDA for eravacycline. Utilizing this revised breakpoint would reduce tigecycline susceptibility prominently to 52.6%, similar to that of eravacycline, proving comparable activity of the two agents which was exemplified by the similarity in MIC distributions of the two agents. Moreover, it is notable that eravacycline demonstrated a superior gastrointestinal safety profile within the tetracycline-glycylcycline class (13) and may be administered once daily at the outpatient setting (14). This information provides a unique place in therapy for eravacycline among the CRE therapeutic options.

The Infectious Diseases Society of America has issued a guidance on the treatment of CRE isolates, recommending BLBLIs as the preferred antibiotics for the treatment of most types of infections since carbapenemase-negative or KPC-producing pathogens prevailed in that region (15). Owing to the relatively high local MBL prevalence, these geographic-specific guidelines are not relevant in Singapore (16, 17). However, our study found that ceftazidime-avibactam inhibited 80 to 90% of non-MBL-producing isolates. The near-universal susceptibility of ceftazidime-avibactam against KPC- and OXA-48-like producing CRE isolates supports its role as an empirical agent where molecular testing results are available. Unlike ceftazidime-avibactam, meropenem-vaborbactam and imipenem-relebactam exhibited variable activity against non-KPC-producers. Meropenem-vaborbactam has been proposed as salvage therapy for ceftazidime-avibactam-resistant KPC infections but has little relevance in our setting currently due to the negligible prevalence of KPC variants (18). While the activity of imipenem-relebactam does not seem to be promising in the treatment of CRE locally, its utility in non-carbapenemase-producing P. aeruginosa, especially those displaying resistance to ceftolozane-tazobactam and/or ceftazidime-avibactam could be further explored.

Interestingly, noncarbapenemase producers had lower BLBLI susceptibility rates (70 to 80%) than KPC producers (>90%). This result is in contrast to the study by Castanheira et al. (19) which demonstrated that all three agents inhibited >93% of the carbapenemase-negative CRE isolates tested. The *in vitro* activity of BLBLIs is likely affected by selective inhibition of certain β-lactamases within strains presenting with multiple enzymes, which may not be manifested when present singly. In our study, nearly all isolates harbored diverse ESBLs or other β-lactamases in various combinations. Furthermore, the three agents studied here are amenable to resistance mediated by porin inactivation and efflux activity (20). Although we did not study the contribution of these mechanisms (e.g., via expression studies using quantitative PCR experiments), it is likely that our isolates were affected by these mechanisms to manifest the levels of the phenotypic carbapenem resistance expressed (MIC_50/90_, 8/≥32 mg/L). Differences in expression levels of the various β-lactamases could also impact the activity BLBLIs. In fact, we postulate that the extent of porin inactivation, efflux, and β-lactamase overexpression is higher in carbapenemase-negative isolates than that in KPC-producing ones, leading to poorer susceptibilities.

With the availability of whole-genome sequencing (WGS) results, we were able to gain better insights into antibiotic susceptibilities in relation to resistance genotypes and to detect emerging resistance trends, providing a perspective on the presumptive utility of novel agents in the region. In our study, variation in resistance genotypes impacted the *in vitro* activity of various antibiotics. For instance, 16S RMTases were detected frequently in CRE isolates with *bla*_OXA-48-like_ and/or *bla*_NDM_. This finding explained the overall low plazomicin susceptibilities in isolates carrying *bla*_OXA-48-like_ and dual carbapenemase producers. An association of 16S rRNA methyltransferases and carbapenemases in *Enterobacterales* has been increasingly reported (21, 22). Although the numbers analyzed here were small, it was also noteworthy that ST231 and ST14 K. pneumoniae accounted for 15 (22.1%) and 14 (20.6%) of 16S rRNA methyltransferases-carrying isolates, respectively. Expansion of these lineages carrying a repertoire of MDR resistance elements could result in dire circumstances. The dissemination of 16S RMTases together with carbapenemases has also been linked to high-risk lineages such as ST147 in Spain (23). The utility of plazomicin could significantly decrease should there be an increase in the prevalence of these resistance determinants.

This study is not without limitations. Our cohort consisted of isolates obtained from a single institution through convenience sampling and may not be representative of all isolates or of those from other institutions in the region. However, the carbapenemase distribution here is largely representative of the local CRE molecular epidemiology (24). This diverse epidemiology encountered in our setting is likely the consequence of the exchange of highly mobile populations resulting from international travel and medical tourism, where major circulating clones encompassed those described in other Southeast Asian/Asian countries. The data generated here could potentially serve to inform policies especially in resource-limited countries lacking comprehensive surveillance data (16, 17, 25). We were also unable to evaluate all recently developed antibiotics (e.g., cefiderocol and aztreonam-avibactam) due to limited access to these drugs under development at the time of the study.

A robust armamentarium of new therapeutic agents for CRE isolates has been introduced into the market in the last few years, lending some promise to the management of infections they cause. For the administration of empirical therapy, a probabilistic approach considering the local ecology should be applied. This approach can be supported by surveillance studies where genomic characterization could contribute to antibiotic decision-making. The lack of harmonization of susceptibility test interpretative criteria also presents a challenge in the comparison of current and old antimicrobial agents. The findings here define the presumptive utility of antimicrobial agents in the region, where plazomicin and ceftazidime-avibactam are promising agents in the management of CRE infections. Nevertheless, continuing antimicrobial resistance surveillance that includes molecular mechanisms is critical for detecting emerging resistance and for defining effective management strategies.

## METHODS

### Bacterial isolates.

A total of 365 unique CRE clinical isolates were randomly selected for testing. This set comprised carbapenem-resistant (defined as nonsusceptibility [intermediate or resistant] to ≥1 carbapenem) isolates collected between 2007 and 2020 for an ongoing carbapenem-resistant Gram-negative pathogen surveillance study (originally obtained from Singapore General Hospital’s Diagnostic Bacteriology Laboratory) and those which were submitted to the Singapore General Hospital Pharmacy Research Laboratory for antibiotic combination testing. The study isolates were representative of the strains frequently encountered in our region, including bacterial isolates collected from nonresidents/foreign patients seeking treatment in Singapore (16, 17).

The bacterial genus and species were identified and confirmed as per the institution’s microbiology laboratory routine procedures, i.e., using Vitek GNI+ cards with the Vitek 2 instrument (bioMérieux, Hazelwood, MO) and/or matrix-assisted laser desorption ionization–time of flight mass spectrometry (MALDI-TOF MS) system (BrukerDaltonik GmbH, Germany). Isolates were preserved in Microbank cryovials (Pro-Lab Diagnostics, Richmond Hill, ON, Canada) at −80°C and subcultured twice on Trypticase soy agar + 5% sheep blood plates (BD, Sparks, MD) before experimental testing.

### Antibiotic susceptibility testing.

MICs for ertapenem, doripenem, imipenem, meropenem, cefepime, aztreonam, piperacillin-tazobactam, levofloxacin, amikacin, tigecycline, polymyxin B, and ceftazidime-avibactam were determined using customized 96-well broth microdilution plates (Trek Diagnostics, East Grinstead, UK); eravacycline (Tetraphase Pharmaceuticals Inc., Waltham, MA) and imipenem-relebactam (Discovery Fine Chemicals, UK, and Valdepharm, Val-de-Reuil, France, respectively) MICs were determined using 96-well broth microdilution plates prepared in-house in accordance with recommendations by Clinical and Laboratory Standards Institute (CLSI) (26). Due to the unavailability of the reference drug powders in Singapore at the time of the study, MICs for meropenem-vaborbactam (bioMérieux, Marcy l’Etoile, France) and plazomicin (Liofilchem, Roseto degli Abruzzi, Italy) were determined using antibiotic gradient test strips. Quality control was conducted using ATCC control organisms as recommended by CLSI or the manufacturer (26, 27).

Avibactam, relebactam, and vaborbactam were tested at fixed concentrations of 4, 4, and 8 mg/L, respectively, in combination with doubling dilutions of their partner β-lactams. MICs of the BLBLIs (ceftazidime-avibactam, imipenem-relebactam, and meropenem-vaborbactam) were determined only for the 274 isolates not possessing metallo-β-lactamases (MBLs) since BLBLIs are not active against MBLs.

Categorical susceptibilities were interpreted according to the 2023 CLSI criteria and the U.S. FDA criteria if CLSI breakpoints were not available (tigecycline and eravacycline) (27). Fosfomycin CLSI breakpoints were applied only to E. coli since existing breakpoints are restricted to E. coli isolates.

### Molecular characterization.

As described elsewhere, whole-genome sequencing was employed for the molecular characterization of the isolates (17, 25).

### Ethics statement.

This retrospective study involved archival bacterial isolates which do not fall under the Human Biomedical Research Act, and no identifiable data were collected. Therefore, the study was exempted from review by the Singhealth Centralised Institutional Review Board.

### Data availability.

The sequences from this study have been deposited in the NCBI Sequence Read Archive (SRA) BioProject under accession no. PRJNA577535.
